# Geriatricians as Primary Care Providers in Payvider Programs: A Strategic Solution

**DOI:** 10.1111/jgs.70413

**Published:** 2026-03-30

**Authors:** Richard G. Stefanacci, Ankur Patel

**Affiliations:** ^1^ Thomas Jefferson University College of Population Health Philadelphia Pennsylvania USA; ^2^ Inspira LIFE Vineland New Jersey USA

**Keywords:** geriatricians, nurse practitioners, payviders

*The geriatrics workforce shortage is real, but the response must be strategic rather than reductive. The main goal of geriatric training should not be to produce primary care providers for fee‐for‐service practice—a system that structurally devalues the comprehensiveness, time, and expertise that geriatric care demands*.

Academic health systems increasingly operate as “payviders”—integrated organizations serving as both healthcare payers and providers. Whether through PACE (Program of All‐Inclusive Care for the Elderly) programs, health system‐owned Medicare Advantage plans, accountable care organizations bearing downside risk, or value‐based care contracts with full capitation, academic medical centers now manage populations under payment models where they assume financial accountability for the total cost of care [1]. This fundamental shift from fee‐for‐service to capitated or risk‐based payment transforms the economic calculus of workforce decisions.

For populations of frail older adults—the fastest‐growing and highest‐cost segment of any payvider's panel—the choice of primary care provider (PCP) model has profound implications. As geriatricians, we face a familiar narrative: our specialty is critically important but economically disadvantaged, our services essential but our workforce shrinking, and our training comprehensive but our salaries lagging. This narrative has led some organizations to question whether geriatrician physicians remain viable, with some replacing them with nurse practitioners (NPs) who command salaries approximately $130,000 lower annually [2].

However, this seemingly logical cost‐saving measure ignores a fundamental principle of capitated care: what matters is not the cost of the provider, but the total cost of care. When analyzed comprehensively—accounting for panel size, specialist utilization, hospitalization rates, and emergency department visits—geriatricians represent not a cost burden but a strategic investment that generates substantial returns for payvider organizations while simultaneously advancing graduate medical education.

## 1 The Total Cost of Care Calculation

The salary differential between geriatricians ($260,000) and NPs ($130,000) represents only the opening chapter of the financial story [2]. Understanding this differential requires accounting for the panel capacity advantage. Research demonstrates that geriatricians can effectively manage panels of 160 participants while NPs manage 80 participants—a 50% capacity advantage applicable across payvider models serving complex older adults [3, 4]. When accounting for panel sizes, the geriatrician cost per patient is $1625 annually—identical to the $1625 for NPs—demonstrating that population‐adjusted staffing costs are equivalent.

The critical cost driver, however, is specialist utilization. Published research consistently demonstrates that NPs refer to specialists at substantially higher rates than physicians: 29% more likely for cardiology, 64% more for endocrinology, and 90% more for nephrology consultations [5]. In payvider models operating under capitation or global budgets, where each specialist consultation includes not just the visit but transportation costs (for PACE or home‐based programs), care coordination overhead, and diagnostic testing, the true cost averages $1000 per consultation.

Using conservative estimates derived from Medicare populations, geriatrician‐managed participants generate approximately 2.5 specialist consultations annually compared to 4.0 for NP‐managed participants—a differential of 1.5 fewer consultations per year [5, 6]. At $1000 per consultation, this represents $1500 per participant per year in avoided specialist‐related costs.

Additionally, geriatricians demonstrate lower emergency department utilization (11.3% fewer visits) [7] and reduced hospitalization rates [8]. ED visit differentials conservatively account for approximately $45 per participant annually. Hospitalization rate reductions represent the most substantial cost driver. For high‐risk populations typical of PACE programs or Medicare Advantage Special Needs Plans, geriatrician‐managed cohorts generally demonstrate 0.10 to 0.20 fewer hospitalizations per patient annually. Using a conservative estimate of 0.15 fewer hospitalizations at an average cost of $13,000 per hospitalization, this differential represents approximately $1950 per participant per year.

Combining these components yields per‐patient annual net cost savings of approximately $3495 (specialist consultation avoidance +$1500; ED reduction +$45; hospitalization reduction +$1950). Remarkably, because population‐adjusted staffing costs are equivalent, these savings represent pure margin improvement. For a payvider program managing 400 high‐risk older adults (a typical PACE program census), this translates to net annual savings of approximately $1,398,000 when choosing geriatricians over NPs as PCPs—achieved at no additional staffing cost. Even under conservative scenarios (0.10 fewer hospitalizations), annual savings exceed $1.1 million per 400 participants.

## 2 Why Geriatricians Reduce Specialist Utilization in Capitated Models

These cost differentials are not merely statistical artifacts; they reflect fundamental differences in training and clinical breadth. Geriatricians complete 8–9 years of post‐undergraduate medical training, including internal or family medicine residency and geriatrics fellowship, developing comprehensive expertise in managing multimorbidity, polypharmacy, and atypical disease presentations [9]. This training enables geriatricians to manage complex conditions that might otherwise trigger specialist referrals: the patient with heart failure, diabetes, and chronic kidney disease who requires integrated medication management rather than three separate specialist consultations; the patient with delirium whose evaluation can be managed in‐house rather than requiring emergency neurology consultation.

Research on integrated geriatric care demonstrates that geriatrician‐led models reduced outpatient specialty visits by 18.18 per year, reduced ED visits, and hospitalizations, and reduced medication burden—with effects most pronounced for patients with high comorbidity burdens [10]. In fee‐for‐service environments, these reductions might harm revenue. In payvider models under capitation or risk‐based contracts, they represent exactly what the payment model rewards: comprehensive, coordinated care that prevents downstream costs.

## 3 The Strategic Value for Academic Health Systems: Graduate Medical Education

For academic health systems operating payvider programs, geriatrician PCPs provide critical infrastructure extending beyond direct clinical and financial benefits. ACGME‐accredited Internal Medicine and Family Practice residency programs require meaningful geriatrics education, including hands‐on clinical experience with frail older adults in diverse care settings. Yet many academic health systems struggle to provide high‐quality geriatrics rotations, particularly in integrated care models that reflect the future of healthcare delivery.

Payvider programs staffed with fellowship‐trained geriatricians create natural rotation sites where residents receive expert supervision in comprehensive geriatric assessment, interdisciplinary team‐based care, and the management of complex multimorbidity in community‐dwelling or institutionalized elders. This serves multiple strategic purposes: geriatrician preceptors provide specialized teaching that residency programs need to meet ACGME geriatrics competencies, potentially avoiding costly external rotation arrangements. Geriatrician PCPs serve simultaneously as clinical providers generating cost savings, residency faculty meeting educational requirements, and academic faculty advancing the institutional mission—without requiring separate GME funding for their teaching role.

Early exposure to high‐quality geriatric medicine in well‐functioning payvider models may increase resident interest in geriatrics careers. If even a small percentage of rotating residents subsequently pursue a geriatrics fellowship, academic health systems can essentially “grow their own” geriatrics workforce. Training residents in payvider settings exposes them to population health management, care coordination, and total cost of care accountability—precisely the competencies needed for future practice in value‐based payment models.

Critically, this educational infrastructure cannot be replicated with an NP‐based PCP model. While NPs serve important roles in resident education, they cannot serve as primary supervisors for physician residency training in geriatrics. The choice of PCP model thus affects not just individual program finances but the institution's capacity to fulfill its educational mission and develop its future physician workforce.

## 4 The Case Against Geriatricians as PCPs—and the Exception That Proves the Rule

Because of the well‐documented challenges to reimbursement for geriatricians in fee‐for‐service Medicare, the main goal of training in and practice of geriatrics should not be to train geriatricians as PCPs. On this point, there is broad consensus, and for good reason. The fee‐for‐service system compensates physicians based on visit volume and relative value units, creating a reimbursement structure that systematically disadvantages geriatricians in three compounding ways.

First, geriatricians' patients require more time. A comprehensive visit addressing polypharmacy, cognitive screening, fall risk assessment, advanced care planning, and functional status—the core elements of the Geriatric 5 M's framework—cannot be accomplished in a 15‐min encounter. Geriatricians routinely require 30–40 min per visit for established patients, reducing daily volume compared to internists managing younger, less complex panels.

Second, geriatricians are paid less per visit. Their patient panels are disproportionately composed of dual‐eligible Medicare‐Medicaid beneficiaries rather than commercially insured patients. In the Camden, New Jersey area (Medicare Locality 01), the reimbursement differential is instructive. For a moderate‐complexity established patient visit (CPT 99214), commercial insurers typically pay $130–$175, while Medicare pays approximately $130–$140 and Medicaid mirrors Medicare rates. For a lower‐complexity visit (CPT 99213), commercial rates run $100–$125 versus Medicare/Medicaid at $90–$110. A geriatrician whose panel is predominantly dual‐eligible receives Medicare as primary payer with Medicaid crossover covering little to none of the remaining coinsurance under lesser‐of policies—effectively eliminating supplemental payment. An internist with a commercially balanced panel captures the higher rates on a substantial proportion of visits.

Third, these factors compound: fewer patients per day multiplied by lower reimbursement per patient produces a revenue gap that no amount of clinical excellence can close under fee‐for‐service. This is the structural reason geriatrician salaries lag behind other internal medicine subspecialties. The payment model punishes precisely what geriatric training teaches: thoroughness, comprehensiveness, and the time‐intensive work of managing multi‐complexity.

There are, however, several other important roles that geriatricians can and should play in value‐based care models beyond direct primary care delivery. Ouslander, Rackman, and Russell described four such roles: educating primary care clinicians and specialists in high‐quality geriatric care, providing consultation and co‐management for complex and costly conditions such as hip fracture, delirium, polypharmacy, and dementia, serving as trained leaders in VBC programs and health systems, and conducting quality improvement initiatives to build the evidence base for management of common conditions in older adults [11]. These roles leverage geriatric expertise as a force multiplier across health systems, and their development should be a central priority for the field. Investment in these roles will, over time, improve care quality and efficiency in ways that generate meaningful cost savings.

However, in specific programs—particularly PACE and similar capitated models as they evolve—geriatricians may be most appropriate to serve in a primary care role. This is not a contradiction of the principle stated above but rather the exception that illuminates it. The reason fee‐for‐service fails geriatricians is not that their skills are wrong for primary care—it is that the payment model is wrong for their skills. Payvider programs operating under capitation invert every structural disadvantage of fee‐for‐service. Under capitation, there are no RVUs. There is no penalty for spending 40 min with a complex patient. There is no revenue loss when the patient is dual‐eligible rather than commercially insured—the capitated payment is the same regardless of original coverage source. And every unnecessary specialist referral, every avoidable hospitalization, every ED visit that could have been managed in‐house represents a cost to the organization rather than a revenue stream.

In these specific environments, the geriatrician's ability to comprehensively manage heart failure, diabetes, chronic kidney disease, depression, dementia, and polypharmacy within a single longitudinal relationship—without fragmenting care across multiple specialists—becomes the most valuable clinical asset a payvider organization can deploy. This is particularly critical for end‐of‐life care. In fee‐for‐service, the final months of life often generate the highest expenditures through aggressive interventions, repeated hospitalizations, and specialist consultations that may not align with patient goals. Geriatricians are trained to navigate goals‐of‐care conversations, implement palliative approaches, coordinate hospice transitions, and manage complex symptom burden—competencies that reduce suffering while simultaneously reducing costs under capitation. An NP encountering a patient with advancing dementia, recurrent aspiration pneumonia, and a family in crisis is more likely to default to hospital transfer. A geriatrician managing that same patient within a PACE program can mobilize the interdisciplinary team, engage the family in goals‐of‐care discussions informed by prognostic understanding, implement comfort‐focused interventions on‐site, and coordinate hospice enrollment when appropriate—avoiding a hospitalization that may produce no benefit and considerable harm.

Moreover, in these capitated programs, the geriatrician PCP role naturally integrates the other four value streams that Ouslander and colleagues identified. The geriatrician in a PACE program educates the interdisciplinary team daily through the rhythm of team‐based practice. They co‐manage complex geriatric syndromes in real time within the program rather than through external consultation. They function as clinical leaders making population‐level decisions about resource allocation, care protocols, and quality metrics. And they identify patterns across their panels that generate quality improvement initiatives addressing each of the Geriatric M's. The payvider PCP role, in these specific settings, is not traditional fee‐for‐service primary care—it is the operational convergence of the broader value proposition for geriatrics.

The data on NP substitution in these settings reinforce this point. When payvider organizations replace geriatricians with NPs based on salary differential alone—without accounting for downstream costs—they are applying fee‐for‐service logic to a capitated payment model. The NP's lower salary is offset by higher specialist referral rates (29% more for cardiology, 64% more for endocrinology, 90% more for nephrology), higher ED utilization, and increased hospitalizations. Under capitation, where the organization bears the cost of every referral and admission, this substitution generates net losses rather than savings.

We therefore advocate for a two‐track strategy for geriatrics. The primary track, consistent with the framework Ouslander and colleagues described, should develop geriatricians as educators, consultants, VBC leaders, and QI investigators across health systems—roles that do not depend on primary care delivery and that multiply geriatric expertise across a far larger patient population than any individual provider could serve. The complementary track should recognize that in specific capitated programs serving the frailest older adults—PACE, institutional Special Needs Plans, and similar models as they develop—the geriatrician PCP role represents a distinct and financially sustainable practice model where comprehensive training translates directly into measurable value. These tracks are not competing visions for the field. They are complementary strategies for different delivery environments, united by the common principle that geriatric expertise generates value when payment models are designed to recognize it.

## 5 Addressing the Counterarguments

Some payvider administrators might argue that team‐based models with NP PCPs supported by consulting geriatricians could achieve similar outcomes at lower cost. However, this model faces several challenges in risk‐bearing environments. Consultant geriatricians typically bill fee‐for‐service, creating financial friction in capitated models. Consultation models fragment care coordination and reduce the real‐time clinical decision‐making that prevents unnecessary external referrals. Research demonstrates that team‐based configurations with a broad provider mix achieve the best outcomes [12]—but this requires physician leadership of the care team, not physician consultation to an NP‐led team.

Others might note that some studies show comparable outcomes between NP and physician primary care. However, these studies typically examine younger, less complex populations or fail to account for the total cost of care, including downstream specialist utilization and hospitalizations [6]. The high‐acuity populations that payviders manage—particularly frail elders with multimorbidity and functional impairment—represent exactly the demographic where geriatrician training provides maximum value and where cost differentials are most pronounced. For academic health systems specifically, the educational argument is dispositive: no alternative staffing model provides the GME infrastructure that geriatrician PCPs deliver.

## 6 A Call to Action for Academic Health Systems

The American Geriatrics Society, academic health systems, and payvider organizations should collaborate to disseminate evidence on the total cost of care benefits of geriatrician PCPs in capitated models, countering the simplistic salary comparison that drives some staffing decisions. Organizations with mature geriatrician‐managed populations should publish their actual hospitalization rate differentials and cost outcomes to strengthen the evidence base. Academic health systems should develop integrated GME strategies that formalize payvider sites as core geriatrics education hubs for IM and FP residents, create geriatrician recruitment pipelines from residency programs to payvider organizations, and expand payvider programs specifically to create educational infrastructure while generating clinical margin.

## 7 Conclusion

The geriatrics workforce shortage is real, but the response must be strategic rather than reductive. The main goal of geriatric training should not be to produce PCPs for fee‐for‐service practice—a system that structurally devalues the comprehensiveness, time, and expertise that geriatric care demands. The field's future lies in developing the full range of roles where geriatric expertise creates value: as educators shaping how all clinicians care for older adults, as consultants and co‐managers for complex conditions, as leaders of value‐based programs, and as investigators building the evidence base for high‐quality geriatric care.

At the same time, we must not overlook the specific settings where geriatricians as PCPs represent the optimal model. In PACE programs, capitated Medicare Advantage plans, and risk‐bearing ACOs serving frail older adults, the payment structure aligns with geriatric competencies in ways that fee‐for‐service never has and never will. In these environments, geriatricians reduce the total cost of care by $2800–$4100 per patient annually at equivalent population‐adjusted staffing cost, provide irreplaceable end‐of‐life care expertise, meet graduate medical education requirements, develop the workforce pipeline, and integrate all four value‐creating roles through a single position.

The evidence is clear: the future of geriatric practice is not in the fee‐for‐service system that has failed our specialty. It is in value‐based models—both the broader roles across health systems and the specific PCP role in capitated programs—that finally reward what geriatricians do best. It is time for academic medicine, payvider organizations, and the geriatrics community to recognize and act on both dimensions of this opportunity.

## Author Contributions

R.G.S. conceived, researched, and wrote this commentary. A.P. contributed to critical revision of the manuscript.

## Funding

The authors have nothing to report.

## Conflicts of Interest

R.G.S. serves as Medical Director of a PACE program within an academic health system. A.P. serves as President of a PACE program within an academic health system.
